# Graph Adaptation Network with Domain-Specific Word Alignment for Cross-Domain Relation Extraction

**DOI:** 10.3390/s20247180

**Published:** 2020-12-15

**Authors:** Zhe Wang, Bo Yan, Chunhua Wu, Bin Wu, Xiujuan Wang, Kangfeng Zheng

**Affiliations:** 1 School of Cyberspace Security, Beijing University of Posts and Telecommunications, Beijing 100876, China; wangxiaozhe@bupt.edu.cn (Z.W.); wuchunhua@bupt.edu.cn (C.W.); binwu@bupt.edu.cn (B.W.); kfzheng@bupt.edu.cn (K.Z.); 2 School of Computer Science, Beijing University of Posts and Telecommunications, Beijing 100876, China; 3 School of Computer Science, Beijing University of Technology, Beijing 100124, China; xjwang@bjut.edu.cn

**Keywords:** relation extraction, domain adaptation, graph convolution network, non-local features

## Abstract

Cross-domain relation extraction has become an essential approach when target domain lacking labeled data. Most existing works adapted relation extraction models from the source domain to target domain through aligning sequential features, but failed to transfer non-local and non-sequential features such as word co-occurrence which are also critical for cross-domain relation extraction. To address this issue, in this paper, we propose a novel tripartite graph architecture to adapt non-local features when there is no labeled data in the target domain. The graph uses domain words as nodes to model the co-occurrence relation between domain-specific words and domain-independent words. Through graph convolutions on the tripartite graph, the information of domain-specific words is propagated so that the word representation can be fine-tuned to align domain-specific features. In addition, unlike the traditional graph structure, the weights of edges innovatively combine fixed weight and dynamic weight, to capture the global non-local features and avoid introducing noise to word representation. Experiments on three domains of ACE2005 datasets show that our method outperforms the state-of-the-art models by a big margin.

## 1. Introduction

The Internet of Things (IoT) is a large-scale paradigm. In the IoT paradigm, devices communicate with each other regardless of their owner. Communication is not only between machines and people, but also between machines [1] and between machines and intelligent objects [2]. The IoT of connected devices includes different fields, such as healthcare, agriculture, smart cities, smart homes, smart grids, automated vehicles, asset monitoring, environmental monitoring, education, industry, etc. [3]. Each field contains a large amount of data information, and the data distribution in different fields is different. At the same time, each of the above domains contains a large number of sensors, actuators, gateways, servers and related end-user applications [1,3,4]. Data collected from interconnected objects or things are used to produce results in different IoT applications. The basic modules of any IoT solution are connected devices, communication networks, services, management, security and applications [1,5]. Among them, the data information of different fields in the Internet of Things is equivalent to the source domain and target domain information in this article. The cross-domain relationship extraction in the IoT will help the collection, mining and classification of data information.

Collecting traffic information from social networks and using it for travel safety are two challenging issues in Intelligent Transportation Systems (ITSs). The transportation network can be monitored through sensor devices and social network data. Currently, ITSs uses sensor devices to monitor all aspects of the transportation network. ITSs can use social network data to support traffic and control management and to check transportation services. The analysis results of these data can help travelers travel safely, solve problems related to traffic congestion, and increase travel in urban areas. However, ITSs may not be able to collect accurate traffic information from these sensors. In addition, in social media, information related to traffic comes with cross-domain text information, which makes the task of transport text mining and classification more difficult for ITSs [6,7,8,9]. This paper takes the relation extraction of cross-domain information as an entry point to explore cross-domain text mining and classification techniques. Relation extraction plays a pivotal role in addressing the issue of information extraction, which aims to detect the semantic relationship between real-world entities [10].

Relation extraction refers to extracting the semantic relation between two candidate entities, i.e., triples such as (United States, Country—President, Trump). In real life, the target domain often has only a small amount of labelled data or even no labelled data; thus, using source domain-labelled data to improve the extraction of relations from the target domain is necessary. However, due to the different data distributions of different domains, directly applying a model trained on the source domain to the target domain results in great performance decreases.

Most works have solved this issue by extracting shared features between the source and target domains and then using these features to perform relation extraction. References [11,12,13] used manually crafted features, such as word clustering and latent semantic analysis, to learn the general representations of different domain words. Due to the limited manual feature space, this method was unable to capture enough shared features for relation extraction. The authors in [14,15] used deep learning methods to project the source features and target features into one unified space and then performed adversarial training to automatically extract the shared features. To avoid introducing domain-specific features, the authors in [16] improved the domain-separated network [17] to extract domain-specific features and domain-independent features separately. However, this method suffered information loss as a result of introducing word embedding reconstruction.

**Figure 1:**
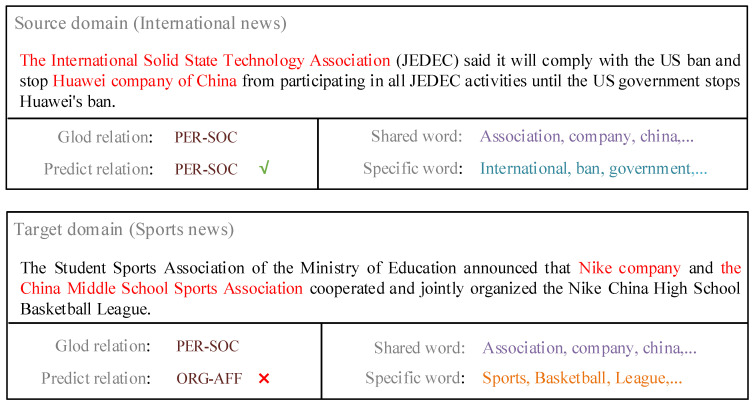
Limitation of traditional methods. Words in red color indicate candidate entities. Shared word indicates the words both appeared in the source domain and target domain. Specific word indicates the words only appeared in the source/target domain.

Other works focused on aligning the domain-specific features between domains. References [18,19] believed that the features gradually transition from being domain-independent to being domain-specific as the depth of the network increases; thus, they proposed deep adaptation networks to align these deep specific features. However, both above methods only enhanced the transferability of the local or sequential features in domain-specific layers while ignoring non-local features. In addition, they applied their ideas in the image field rather than relation extraction. In some cases, non-local features, such as word co-occurrences and co-references, are also significant for cross-domain relation extraction. Figure 1 shows an example under this situation. The source domain data and target domain data have the same gold relation PER-SOC, but the contexts near entities are very different between the two domains; specifically, the domain-specific words have strong domain relevance. The traditional local-feature-based models cannot align these specific features because they are semantically irrelevant; as a result, they obtain the wrong relation ORG-AFF. On the other hand, it can be observed that some specific words are often co-occurring with shared words. According to [20], the more times these specific words appear together with shared words, the greater they should be aligned. Reference [20] aligned domain-specific words with a bipartite graph based on shared and specific word co-occurrences. This approach used spectral clustering to reduce the gap between specific features across domains. However, these manually crafted features are too limited to scale.

To solve the problems mentioned above, inspired by [20], we propose an end-to-end graph adaptation network to adapt non-local features between domains. Graph structures effectively capture non-local features in relation extraction [21,22], but no studies have applied graph structures to cross-domain relation extraction, the edges in the proposed graph only exist between specific words and shared words. In this paper, source domain words and target domain words indirectly connect through shared words as intermediate agents and word co-occurrence information as the fixed weights of edges. However, word co-occurrence information has a strong dependence on the corpus and inevitably introduces some noise; therefore, we propose a method to calculate the dynamic weights of the edges using the attention mechanism. The fixed weights and dynamic weights are combined as the final edge weights.

The word information is propagated through graph convolutions on the tripartite graph so that the domain-specific word representations are aligned. After adapting the non-local features, i.e., obtaining the aligned word representations by the graph adaptation network, we perform adversarial training to extract the shared local features between domains. These word representations are aligned before being fed into downstream modules; therefore, the model can extract shared features effectively. Finally, the shared features are fed to a fully connected layer to perform relation extraction. Because we assume that only source domain data have labels, the relation classifier is trained using only source-labelled data. In addition, to prevent the transfer of irrelevant information from words, we select valuable edges and remove irrelevant edges depending on their fixed weights. Using this method, the model not only preserves useful information for adapting non-local features but also speeds up the calculation process.

The major contributions of our work are as follows:

- We propose a novel graph adaptation network to align domain-specific features. Local features and non-local features are transferred simultaneously for cross-domain relation extraction. This is also the first work to adapt the non-local features between domains.
- Unlike the traditional graph convolutional network, our network combines fixed weights and dynamic weights as the edge weights of the graph. In addition, rather than using a fully connected graph, we only keep valuable edges based on their edge weights. These strategies can transfer useful information effectively and avoid introducing irrelevant noise.
- Experiments have shown that non-local features such as word co-occurrences are also important for cross-domain relation extraction. The proposed method to calculate weights and select edges can capture more non-local features and better avoid noise interference than other methods.

The rest of this paper is organized as follows. Section 2 presents the work carried out in the field of cross-domain relation extraction. Section 3 introduces the task definition of the relation extraction. Section 4 describes a graph adaptation network for cross-domain relation extraction. Section 5 presents experiments that analyze the effectiveness of our model. Section 6 concludes and presents future work.

## 2. Related Work

Data collection and data query are important applications in wireless sensor networks (WSN) and the IoT, and data collection and data query are usually information-centric. We noticed that WSN is a special network, in which each sensor will involve sensor data in different fields (for example, smart cities, healthcare, agriculture, etc.). Each field contains a large amount of data information, and the data distribution in different fields is different. In the last decade, deep learning (DL) has made breakthroughs in natural language processing (NLP), image processing and reinforcement learning, so that making breakthroughs in the field of artificial intelligence (AI) occupy the dominant position. Presently, much research contributed to information mining and the utility of massive data [23]. In addition, Lei et al. used multi-sensor data in fault detection of gearbox [24]. Safizadeh et al. studied multi-sensor data fusion to improve the performance of fault recognition for rolling element bearings [25]. Jing et al. combined deep neural networks and multi-sensor data fusion in the fault detection of planetary gearbox [26]. Compared with these fields, DL methods for cross-domain data analysis of sensors are relatively scarce [27]. At the same time, few papers researched cross-domain deep feature learning and fusion models. However, the representativeness of common features will obviously affect the ability of cross-domain information mining and classification. These problems encourage researchers to find a new method to adaptively extract cross-domain relationships, which in turn helps to mine and classify information in different fields.

Cross-domain relation extraction aims to solve the problem of the training set and test set with different data distributions. Reference [11] was the first work to adapt a relation extraction model to other domains, and it used generalized approaches such as word clustering to extract shared features. The authors in [13,28] combined hand-crafted features, such as dependency paths and learned word embeddings, for cross-domain relation extraction. These methods use manually crafted features to adapt existing features, so they are limited and lose some information. The deep learning approach was applied to cross-domain relation extraction in [29]. It combined feature-based methods and neural networks to exploit their advantages. Reference [15] used adversarial training to extract shared features by introducing a gradient reversal layer (GRL) [30], but it simply projected source features and target features into one unified space, inevitably introducing some domain-specific features that harmed the performance of extracting relations with the target domain. Reference [16] proposed a genre separation network to extract shared features and specific features separately. Reference [31] applied cross-view training [32] to a domain adversarial neural network (DANN) [15] and adapted shared features in different views; this is a highly fine-gained method.

Massive domain adaptation methods were also applied in other tasks. Reference [20] used spectral clustering to unify specific word representations in the context of text classification. Reference [33] used some labelled data from the target domain for learning domain-specific information. Reference [34] aligned different domain cells of the sequence model to perform domain adaptation; this is another fine-grained method. In the image field, [18,19] aligned deep specific features using distance metrics such as the maximum mean discrepancy (MMD). These methods provided us with inspiration to align domain-specific features for the task of cross-domain relation extraction task, but they only transferred local features while ignoring non-local features. Although [20] aligned specific features by using non-local features such as word co-occurrence information, complex feature engineering was needed.

Recently, graph structures were widely used in natural language processing tasks to capture non-local features. Reference [21] applied graph convolutions to pruned dependency trees and automatically captured the dependence information. Reference [35] used a graph convolutional network to model the co-referent and identical mentions between words. Reference [22] combined linear and dependency structures to improve the extraction of overlapping relations. Reference [36] proposed an entity-relation graph to perform joint type inference on entities and relations and used the entity-relation bipartite graph in a highly efficient and interpretable way. Reference [37] proposed a graph-based method to improve word embeddings. Reference [38] used graph neural networks with generated parameters to improve multi-hop reasoning. To specify the weights of neighbors automatically without requiring any kind of costly matrix operation or depending on knowing the graph structure upfront [39], the authors in [39,40] introduced an attention mechanism for the graph structure. These studies inspire us to use a suitable graph structure to model cross-domain relation extraction problems.

## 3. Task Definition

### 3.1. Relation Extraction

Given a set of labeled corpus $D=\{\left(s_{1},e_{11},e_{12},r_{1}\right),\ldots ,(s_{n},$$e_{n1},e_{n2},r_{n})\}$, where $e_{i1}$ and $e_{i2}$ (*i* = 1, 2, …, *n*) denote the first and second candidate entity respectively, $r_{i}$ represents the relation type, $s_{i}$ represents a sentence, relation extraction can be regarded as a classification task that applying a classifier *f* trained on *D* to the test datasets $D^{{}^{\prime }}=\left\{\left(s_{1}^{{}^{\prime }},e_{11}^{{}^{\prime }},e_{12}^{{}^{\prime }}\right),\ldots ,\left(s_{n}^{{}^{\prime }},e_{n1}^{{}^{\prime }},e_{n2}^{{}^{\prime }}\right)\right\}$. In other words, considering the task where *X* is the input space and *Y* is the set of relation labels, the goal of the learning algorithm is to build a classifier *f*:X→Y with a low loss $L\left(D^{’}\right)=E_{\left(s^{’},e_{1}^{’},e_{2}^{’},r^{’}\right)∼D^{’}}P\left(f\left(s^{’},e_{1}^{{}^{\prime }},e_{2}^{{}^{\prime }}\right)\neq r^{’}\right))$.

### 3.2. Cross-Domain Relation Extraction

Given a set of source labeled corpus $D_{s}=\{\left(s_{1},e_{11},e_{12},r_{1}\right),$$\ldots ,(s_{n},$$e_{n1},e_{n2},r_{n})\}$ and target unlabeled corpus $D_{t}=\left\{\left(s_{1}^{{}^{\prime }},e_{11}^{{}^{\prime }},e_{12}^{{}^{\prime }}\right),\ldots ,\left(s_{n}^{{}^{\prime }},e_{n1}^{{}^{\prime }},e_{n2}^{{}^{\prime }}\right)\right\}$, the meanings of these symbols are the same as Section 3.1. It is worth noting that we assume that there is no labels in target domain data. Then cross-domain relation extraction can be regarded as a classification task that uses source domain labeled data $D_{s}$ and target domain unlabeled data $D_{t}$ to train a classifier *f*, and applies *f* to target domain. The goal of the learning algorithm is to build a classifier *f*:X→Y with a low loss $L\left(D_{t}\right)=E_{\left(s^{’},e_{1}^{’},e_{2}^{’},r^{’}\right)∼D_{t}}P\left(f\left(s^{’},e_{1}^{{}^{\prime }},e_{2}^{{}^{\prime }}\right)\neq r^{’}\right))$.

## 4. Our Methodology

In our work, we take $D_{s}$ and $D_{t}$ as inputs to design an algorithm that can improve the extraction of relations from the target domain. First, $D_{s}$ and $D_{t}$ are fed into an embedding layer to obtain an embedding matrix, and then the graph convolutions work on the embedding matrix to align domain-specific word representations. A feature extractor takes the adjusted embedding matrix as input to obtain the shared features. To force the feature extractor to extract these shared features, a domain discriminator is added after the feature extractor. Finally, the shared features are fed into a relation classifier to perform classification.

**Figure 2:**
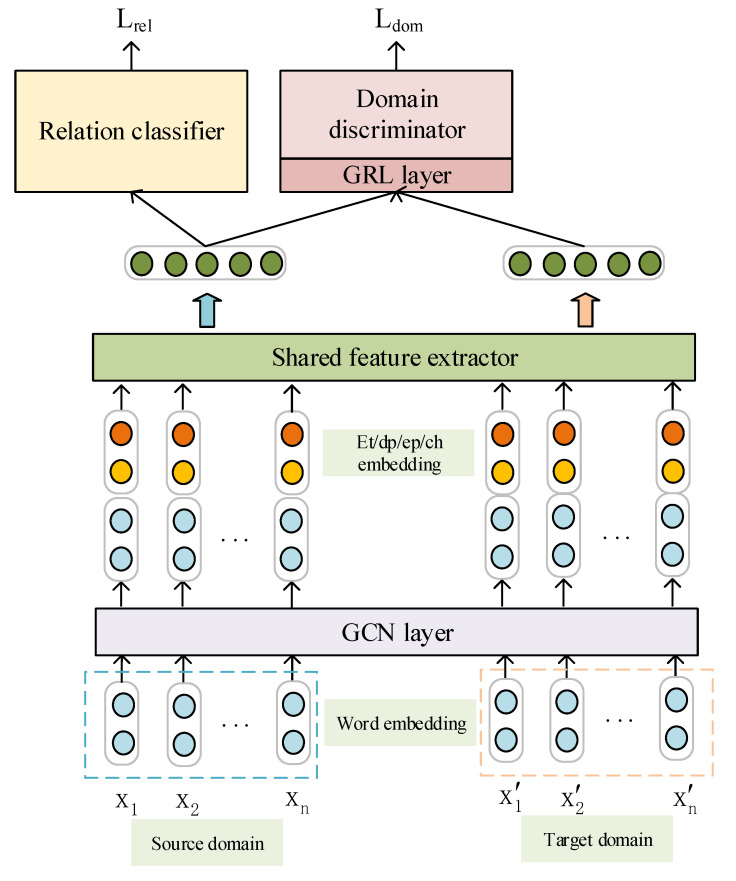
The overall architecture of our graph adaptation network.

In brief, our model consists of four modules: an adaptation module, an embedding layer, a shared feature extractor and a relation classifier. The adaptation module contains a domain discriminator and a graph convolutional network (GCN) layer, which are responsible for local shared feature extraction and non-local feature alignment, respectively. Figure 2 shows the overall architecture of our model. We introduce these modules in detail below.

### 4.1. Adaptation Module

The adaptation module mainly contributes to extracting the shared features between domains and aligning domain-specific features. The traditional approach only adapts sequential features but ignores non-sequential or non-local features. Our adaptation module consists of two processes: local information adaptation and non-local information adaptation. Local information adaptation is applied at the sentence level; in other words, we extract the shared features of the source and target sentences. While non-local information adaptation is a word-level adaptation, it uses a GCN to align domain-specific word representations. The remainder of this section elaborately introduces the adaptation layer.

(1) Local information adaptation (sentence-level).

To make the shared feature extractor capture domain-invariant features, a domain discriminator is added after the shared feature extractor. It takes $s_{s}$ and $s_{t}$ as inputs, where $s_{s}$ and $s_{t}$ represent the source and target features extracted by the shared feature extractor, respectively. The domain discriminator is implemented by a simple neural network with one hidden layer and performs binary classification to predict the domain that a sample comes from. The domain discriminator loss is defined as cross entropy loss:

(1) $$L_{dom}=-\frac{1}{N_{s}+N_{t}}\sum_{i=1}^{N_{s}+N_{t}}\left(1-y_{i}\right)log\left(1-p_{i}\right)+y_{i}logp_{i}$$

In this equation, $N_{t}$ denotes the total number of target domain data, $p_{i}$ is the probability of one sample belonging to the source domain and $y_{i}\in \left\{0,1\right\}$ indicates that the sample comes from the source domain (1) or the target domain (0).

To confuse the domain discriminator, a gradient reversal layer (GRL) [30] is used between the shared feature extractor and domain discriminator. Then, the forward and back propagations are formulated as follows:

(2) GRL(x)=x

(3) $$\frac{d(GRL(x))}{dx}=-I$$

Through reversing the gradient before domain discriminator, the parameters of domain discriminator are optimized to reduce the domain discriminator loss $L_{dom}$ while the parameters of shared feature extractor will make $L_{dom}$ increase. The adversarial training finally converged so that the discriminator cannot distinguish which sample comes from which domain, in other words, the shared feature extractor captures some domain-invariant features.

(2) Non-local information adaptation (word-level).

**Figure 3:**
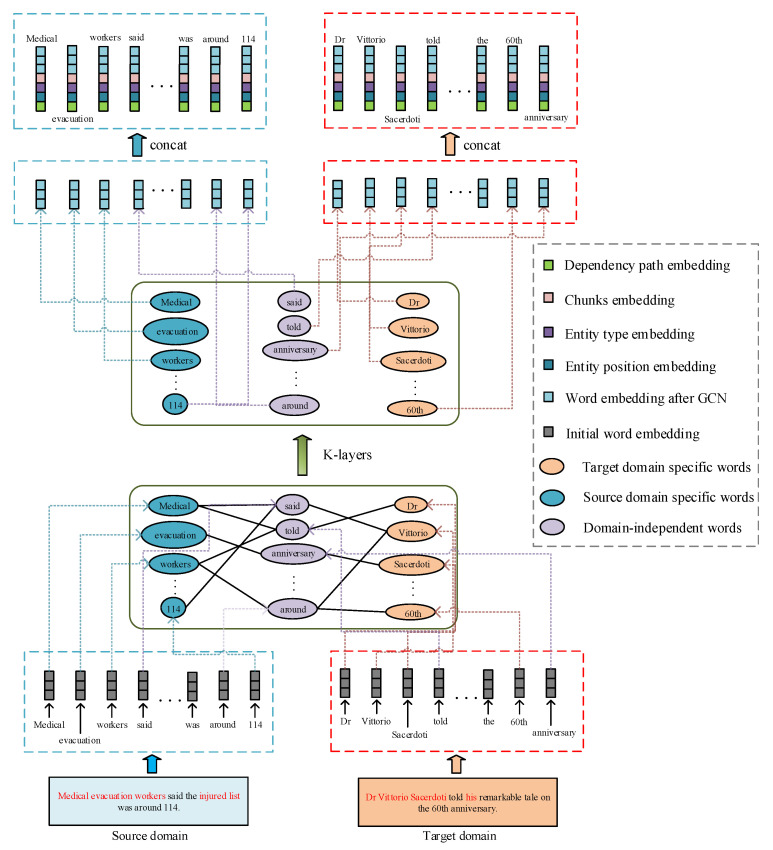
The architecture of GCN layer for aligning domain-specific words.

Word-vectorized representations, such as word2vec [41] and Glove [42], have greatly improved downstream applications. However, in cross-domain relation extraction, the representations of domain-specific words differ significantly between domains, and this causes poor performance when applying a model to other domains. Most previous works only focused on aligning different domain features at the sentence level [15,16,19] while ignoring word-level alignment. Inspired by [20], rather than using feature-based methods, we use a GCN to model the word co-occurrences of different domains. Through this alignment, the word representation gap between the source domain and target domain can be reduced, thereby enabling the downstream module to extract the shared features in a fine-grained way. Figure 3 shows the architecture of the GCN layer.

Word co-occurrence tripartite graph construction: The key idea of non-local information adaptation is, in the tripartite graph, if two domain-specific words have connections to more common domain-independent words in the graph, they tend to be aligned together with higher probability, i.e., have similar word representation [20]. Given the source domain sentence set $D_{s}$ = $\left\{S_{1},S_{2},\ldots ,S_{n}\right\}$ and target domain sentence set $D_{t}=\left\{S_{1}^{{}^{\prime }},S_{2}^{{}^{\prime }},\ldots ,S_{n}^{{}^{\prime }}\right\}$, we construct a graph *G* = $\left(V_{s}\cup V_{i}\cup V_{t};E_{si}\cup E_{ti}\right)$ for any two sentence $S_{i}=\left\{w_{1},w_{2},\ldots ,w_{n}\right\}$ and $S_{j}^{{}^{\prime }}=\left\{w_{1}^{{}^{\prime }},w_{2}^{{}^{\prime }},\ldots ,w_{n}^{{}^{\prime }}\right\}$. Here, $V_{s}$, $V_{i}$, $V_{t}$ denote the graph vertex that corresponds to domain-specific words in $S_{i}$, domain-independent words in $S_{i}\cup S_{j}^{{}^{\prime }}$ and domain-specific words in $S_{j}^{{}^{\prime }}$ respectively, $E_{si}$ represents the graph edges between $V_{s}$ and $V_{i}$, $E_{ti}$ represents edges between $V_{t}$ and $V_{i}$. See Figure 3 for details.

We use two types of weights for the graph edges: fixed weights and dynamic weights. First, the pointwise mutual information (PMI) of two words is used as a fixed weight. The PMI is an algorithm for calculating the correlation between two variables. Here, we use the PMI to measure the co-occurrence relationship of two words:

(4) $$A_{i\neq j}=log\frac{p(w_{i},w_{j})}{p\left(w_{i}\right)p\left(w_{j}\right)}$$

(5) $$p\left(w_{i},w_{j}\right)=\frac{\#win(w_{i},w_{j})}{\#win}$$

(6) $$p\left(w_{i}\right)=\frac{\#win(w_{i})}{\#win}$$

where $\#win(w_{i},w_{j})$ refers to the number of sliding windows that $w_{i}$ and $w_{j}$ appeared together. #win is the total number of sliding windows. We calculate these on the whole corpus. A higher PMI value means that $w_{i}$ and $w_{j}$ appear together on the corpus more times; thus, they have a higher correlation. A small PMI value means there is little correlation between $w_{i}$ and $w_{j}$ because they seldom appear together. After we obtain the weight matrix $A\in R^{m*m}$, (*m* is the length of the dictionary), the weights are normalized by:

(7) $$f_{i\neq j}=\frac{A_{ij}-a}{b-a}$$

where *a* is the minimum element of $A_{i\neq j}$ and *b* is the maximum element of $A_{i\neq j}$. Then the fixed weights are defined as:

(8) $$F_{ij}=\left\{\begin{array}{cc} f_{ij}, & \;\;i\neq j\&f_{ij}>\alpha \\ 1, & \;\;i=j\\ 0, & \;\;others \end{array}\right.$$

The graph only keeps edges with $f_{ij}>\alpha$, where α>0 is a hyperparameter. When α increases, there will be fewer edges in the graph. The effects of different values of α are discussed in the experiment later in this paper.

Fixed weights capture the word co-occurrence features, but they have difficulty aligning domain-specific words. There are two limitations of using fixed weights: (1) some English stop words such as “*is*” and “*the*” are often domain-independent, and they have a high probability of appearing together with domain-specific words; therefore, the fixed weight between them is large. However, these stop words have little semantic meaning and harm the word representations. (2) Some domain-specific words are rare and seldom appear together with domain-independent words, so the fixed weight is almost 0, but these words should probably also be aligned; fixed weights cannot achieve this.

To compensate for the limitations of fixed weights, inspired by [39], attention mechanism-based dynamic weights are used. First, to increase the power of the feature expressions, a linear transformation is applied on every node in the graph:

(9) $$h_{i}=w_{l}h_{i}+b_{l}$$

where $h_{i}\in R^{n}$ is the vector representation of node *i*, $w_{l}\in R^{n*n}$ and $b_{l}\in R^{n}$ is the parameters of linear transformation. For a node *i* in graph, N(i) is defined as the nodes which directly connect to the node *i* and ∀j∈N(i), $f_{ij}>\beta$. We set β=0.3 for balancing computational efficiency and model effectiveness. Then we calculate the attention weight on N(i) for every node *i*:

(10) $$e_{ij}=LeakyReLU\left(h_{i}^{T}W_{att}h_{i}\right)$$

where $W_{att}\in R^{n*n}$ is the attention parameter and j∈N(i), $e_{ij}$ is the attention weight that indicates how important the node *j* to node *i*, LeakyReLU is an activate function. The dynamic weights of node *i* are normalized by softmax function:

(11) $$D_{ij}=\frac{exp(e_{ij})}{\sum_{j\in N(i)}exp\left(e_{ij}\right)}$$

Dynamic weights can be adjusted during training to provide a flexible way to train the parameters of the model. In addition, each fixed weight can be seen as a global weight because it is calculated based on statistics of the whole corpus, while a dynamic weight can be seen as a local weight because it only uses two sentences per calculation. In the end, we combine the fixed weights and dynamic weights as our final graph weights:

(12) $$W_{ij}=F_{ij}+D_{ij}$$

**Figure 4:**
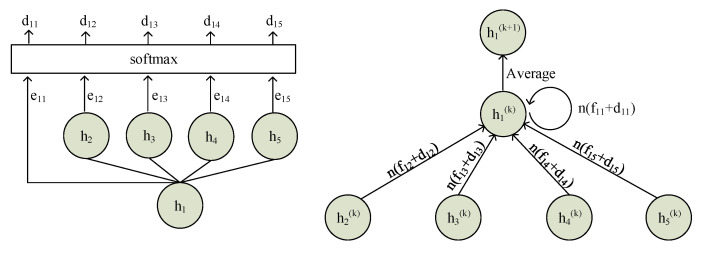
(**Left**): the process of calculating the dynamic attention weights. (**Right**): the information aggregation process. n(·) denotes a normalized function.

The process of calculating the edge weights is illustrated in Figure 4 (left).

Graph convolutions on the tripartite graph: In this section, we first introduce the graph convolution operation and edgewise gating mechanism and then elaborate on how these methods are used in our model.

A GCN [43] is used to capture non-local and nonsequential information. Specifically, given a graph G=(V,E), where *V* is the node set and *E* is the edge set, the graph convolution operation is applied on every node and propagates information to other nodes along the edges. For a 1-layer GCN, the information only transfers to neighboring nodes, and the information of an *n*-layer GCN can transfer to further nodes as *n* increases. The information propagation from layer *k* to layer k+1 can be formulated as:

(13) $$h_{i}^{\left(k+1\right)}=\frac{1}{d(i)}\sum_{j\in N(i)}W_{ij}\left(w_{g}^{\left(k\right)}h_{j}^{\left(k\right)}+b_{g}^{\left(k\right)}\right)$$

where $d\left(i\right)=\sum_{j\in N_{(}i)}W_{ij}$ is sum of all the weights between node *i* and its neighbors. $w_{g}^{\left(k\right)}\in R^{m*m}$ and $b_{g}^{\left(k\right)}\in R^{m}$ are layer specific parameters. $h_{j}^{\left(k\right)}\in R^{m}$ is vector representation of node *j*. Figure 4 (right) gives visual representation of the information propagation.

Edge-wise gate [44] is proposed to control how much information is transferred from neighbors. The scalar gate value of each neighbor is calculated as:

(14) $$g_{j}^{\left(k\right)}=\sigma \left(w_{e}^{\left(k\right)}h_{j}^{\left(k\right)}+b_{e}^{\left(k\right)}\right)$$

Here $w_{e}^{\left(k\right)}\in R^{m}$ and $b_{e}^{\left(k\right)}\in R$ are layer specific parameters, and σ is a non-linear activate function.

According to [37], we integrated edge-wise gating mechanism into the graph convolution network, the final propagation function is:

(15) $$h_{i}^{\left(k+1\right)}=\frac{1}{d(i)}\sum_{j\in N(i)}W_{ij}g_{j}^{\left(k\right)}\left(w_{g}^{\left(k\right)}h_{j}^{\left(k\right)}+b_{g}^{\left(k\right)}\right)$$

when k=0, $h_{j}^{\left(0\right)}=v_{j}$ is the input of GCN, i.e., the word original representation. For a *n*-layer GCN, $h_{i}^{\left(n\right)}$ is the word final representation after the non-local information adaptation.

### 4.2. Embedding Layer

External knowledge, such as entity positions and dependency trees, is important for relation extraction [45,46,47]. Furthermore, when adapting a model from a source domain to a target domain, external knowledge can be seen as general knowledge that can improve cross-domain task performance. Following previous works [15,28,33], we use the following five types of external knowledge:

Real-valued word embedding vector. We obtain one word’s embedding vector $e_{i}$ from the word embedding matrix, which is pretrained as in [41]. This process yields continuous vector representations of words by training the CBOW or skim-gram model on very large data sets, and the vectors include the words’ semantic information. The words that do not exist in the word embedding matrix are randomly initialized.

Words’ relative distances from candidate entities. Use *i* and *j* denote the two entities’ in a sentence, for each word $x_{k}$ with index *k*, its relative distances are k−i and k−j, respectively. The word’s relative distances can inform the model of the entities’ positions. Every word has two relative distances vectors $d_{1}$ and $d_{2}$.

Entity type. Each entity type is predefined, and every entity has an entity type to which it belongs. In the sentence “*He will blow* ***a city*** *off* ***the earth*** *in a minute if he can get the hold of the means to do it*”, the bold words are candidate entities, and their entity types are GPE and LOC. Entity types are essential knowledge for relation extraction. In some cases, we can infer the relation of two candidate entities only by the entity types. In our setting, we only indicate that the entity types and nonentity words are randomly initialized in the same vector. Every word has two entity type vectors $t_{1}$ and $t_{2}$ because we have two candidate entities per sentence.

Sematic chunks. A chunk is an indivisible fixed phrase in a sentence, and we inform the model to regard all chunks as a whole so that the semantic information of the chunks is not disrupted. We use the B-I-O format to indicate chunks, and every word obtains a chunk vector $c_{i}$.

**Figure 5:**
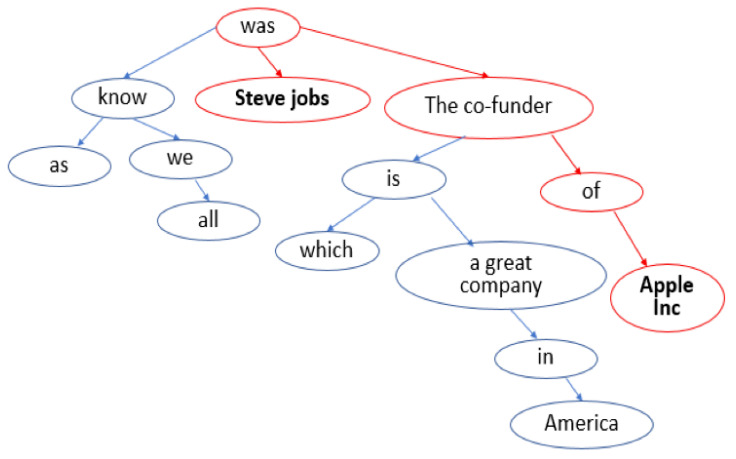
Dependency tree of the sentence “*As we all know, Steve Jobs was the co-founder of Apple Inc. which is a great company in America*”. Bold words are two candidate entities, red lines indicates the shortest dependency path between the two entities.

Shortest dependency path between two entities. The shortest dependency path refers to the shortest path between two entities in the dependency tree. See Figure 5 for an example. In relation extraction, the information required to assert a relationship between two entities is mostly captured by the words in the shortest dependency path between the two entities [45]. Therefore, the shortest dependency path can help the model to distinguish between valuable information and noise. We use a vector $d_{i}$ to indicate whether a word is in the shortest dependency path between two entities.

After getting all above types of embedding vectors, we transform every word into a real-valued vector $v_{i}$ by concatenating them: $v_{i}=\left[gcn\left(e_{i}\right);p_{i1};p_{i2};t_{i1};t_{i2};c_{i};d_{i}\right]$. The gcn(·) is the transformation of GCN layer described in Section 4.1, see Figure 3 for details. The whole sentence with length *n* can be represented as *v*$=[v_{1},v_{2},\ldots ,v_{n}]$.

### 4.3. Shared Feature Extractor

We use a simple CNN architecture proposed by [48] as shared feature extractor. Let $v_{i}\in R^{d}$, and a convolution operation with a kernel $w\in R^{rd}$ is applied to *v*, where *r* is numbers of words the kernel spanned and *v* is the word representation matrix after GCN layer. A feature $c_{i}$ is generated from words $v_{i:i+r-1}$:

(16) $$c_{i}=ReLU\left(wv_{i:i+r-1}+b\right)$$

Here, ReLU is the activation function and b∈R is a bias. The kernel moves one step at a time in the direction of word sequences and get n−h+1 features totally:

(17) $$c=[c_{1},c_{2},...,c_{n-r+1}]$$

Then we perform max-over-time pooling operation [49] on *c*, i.e., $c^{{}^{\prime }}=max\left\{c\right\}$, to get the most important feature. To capture various features, we use multiple kernel size (keep *d* unchanged and use different *r*) and each kernel size has multiple feature maps. For a fixed kernel size, different feature maps will get different $c_{i}^{{}^{\prime }}$, so the feature vector $c_{i}^{{}^{\prime }}$ corresponding to one kernel size *i* is:

(18) $$c_{i}^{{}^{\prime }}=\left[c_{1}^{{}^{\prime }},c_{2}^{{}^{\prime }},\ldots ,c_{m}^{{}^{\prime }}\right]$$

*m* is the number of feature maps. Finally, we concatenate all $c_{i}^{{}^{\prime }}$ to get shared feature extractor’s output $s=[c_{1}^{{}^{\prime }};c_{2}^{{}^{\prime }},\ldots ,c_{k}^{{}^{\prime }}]$, *k* is the number of kernel size.

### 4.4. Relation Classifier

Since there only exists labels in the source domain, the relation classifier only takes the shared features $s_{s}$ from source domain as input to perform relation classification. The relation classifier is a 2-layer fully connected neural network *h* with tanh as activation function and followed by a softmax layer:

(19) $$p_{i}=softmax\left(h\left(s_{s};\theta_{s}\right)\right)$$

where $\theta_{s}$ is the parameters of the hidden layer. $p_{i}\in R^{r}$ is the relation distribution of *i*-th source domain data, and *r* is the number of relation type. The relation classify loss $L_{rel}$ is defined as below:

(20) $$L_{rel}=-\frac{1}{N_{s}}\sum_{i=1}^{N_{s}}\sum_{j=1}^{r}y_{ij}logp_{ij}$$

Here $N_{s}$ is the total number of source domain data, $y_{ij}\in \left[0,1\right]$ to indicate whether the example *i* has relation *j*. $p_{ij}$ is obtained through softmax layer and indicates the probability of example *i* containing the relation *j*.

During training, we combine all the losses mentioned above to get the final loss function and optimize jointly:

(21) $$L_{loss}=L_{rel}+\gamma L_{dom}$$

γ is a hyparameter and we set γ=0.1 through validation dataset. In the test stage, due to lacking of source domain data, a heuristic algorithm is designed to select domain-specific words of source domain. First, for every shared word $w_{i}$ appearing in one target domain sentence, we find the source domain-specific word $w_{j}$ which have the highest PMI value with $w_{i}$. All the $w_{j}$ consist of the top-pmi set $w=\{w_{1},w_{2},\ldots ,w_{n}\}$ which *n* is the number of shared word appeared in the target domain sentence. Then we sort the elements of *w* in descending order. Finally, the top *m* words are selected to form the source domain sentence and we set m=10 through the performance in validation dataset.

## 5. Experiments

### 5.1. Dataset and Evaluation

(1) Dataset

We conduct our experiment on the English part of the ACE2005 dataset. There exist 6 domains (broadcast news (bn), broadcast conversations (bc), newswire (nw), weblog (wl), usenet (un), and conversational telephone speech (cts)) and 7 unidirectional relations (artefact (ART), gen-affiliation (GEN-AFF), org-affiliation (ORG-AFF), part-whole (PART-WHOLE), person-social (PER-SOC), physical (PHYS) and none (METONYMY)). If we consider the directions of the entity pairs, there are 11 directional relations (METONYMY, PER-SOC and PHYS are symmetric relations). We process the datasets as in [28] (Table 1).

**Figure 6:**
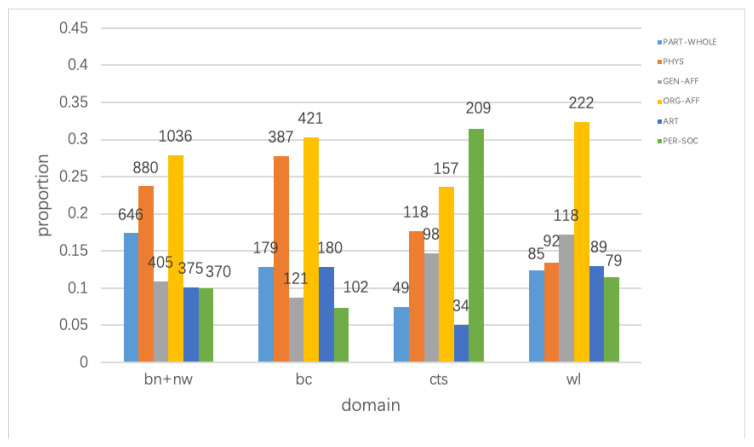
Relation distributions of different domains. Note that we ignore the NONE type because it accounts for a large portion in all three domains.

From Table 1, we can see that negative examples account for a large proportion of the data, so correctly distinguishing between positive and negative examples is an important indicator for measuring the effect of the model. Figure 6 explicitly displays the differences between domains in terms of their data distributions. These large differences also pose a challenge for our model. Following previous works [15,16,33], we use bn+nw as the source domain, adjust the hyperparameter on half of the bc domain, and use the remaining half of bc, as well as all of cts and wl, as the target domain to evaluate the performance of our model.

(2) Evaluation

The precision, recall and macro-F1 are used as evaluation method in our experiment. Specifically, we calculate precision ($P_{i}$) and recall ($R_{i}$) for every relation type, and get all relation precision (*P*) and recall (*R*) by averaging:

(22) $$P=\frac{1}{r}\sum_{i=1}^{r}n_{i}P_{i}$$

(23) $$R=\frac{1}{r}\sum_{i=1}^{r}n_{i}R_{i}$$

where *r* is the number of relation type and $n_{i}$ is the number of samples belong to *i*-th relation. The macro-F1 is calculated using:

(24) $$\mathrm{macro}-F1\;=2\times \frac{P\times R}{P+R}$$

### 5.2. Parameter Setting

We use pretrained, 300-dimensional word embeddings generated by word2vec [11]. The dependency entity positions (eps) are obtained from *ace-data-prep* (https://github.com/mgormley/ace-data-prep). The embeddings are randomly initialized and optimized during training except for these pretrained word embeddings because we found that there is no improvement when pretraining all embeddings. All the sentences are padded or cut to 155 characters. The learning rate is set to 0.001 and halved every 4 epochs. We use Adam [50] as the optimizer and apply gradient clipping during optimization. To avoid overfitting, the dropout technique [51] is used in the embedding layer and GCN layer. The details of the parameter settings are shown in Table 2.

### 5.3. Baseline Models

We use the following baseline models for evaluation purposes:

NNM & log-linear model: Basic neural network models (NNM), such as CNNs and RNNs, were used in [29] to improve relation extraction. In addition, the authors achieved state-of-the-art performance by stacking these models. We use their single model bidirectional RNN (BRNN), CNN, log-linear model, and combined model (called hybrid-voting system (HVS)) as our baseline models.

FCM & hybrid FCM: The feature-rich compositional embedding model (FCM) was proposed in [28]. The key idea is to combine (unlexicalized) hand-crafted features with learned word embeddings. The hybrid FCM (HFCM) combines the basic FCM and existing log-linear models.

LRFCM: The low-rank approximation of the FCM (LRFCM) [13] is an improvement of the FCM. It replaces manual features with feature embeddings so that it can easily scale to a large number of features.

DANN: The domain adversarial neural network (DANN) [15] was the first to introduce adversarial training to cross-domain relation extraction. It simply projects source domain features and target domain features into one unified space and uses adversarial training to extract domain-independent features. We propose a graph adaptation network based on this model.

GSN: The genre separate network (GSN) [16] uses a domain separate network [17] to extract domain-independent features and domain-specific features separately, therefore avoiding the introduction of some domain-specific features into the shared feature space.

CVAN: The cross-view adaptation network (CVAN) [33] uses cross-view training to extract shared features from different views and constructs various input views that have proven to be useful for cross-domain relation extraction.

AGGCN: The attention guided graph convolutional networks (AGGCN) [52], a novel model which directly takes full dependency trees as inputs.This model can be understood as a soft-pruning approach that automatically learns how to selectively attend to the relevant sub-structures useful for the relation extraction task.

MAPDA: A novel model based on a multi-adversarial module for partial domain adaptation (MAPDA) is proposed in this study [10]. This paper design a weight mechanism to mitigate the impact of noise samples and outlier categories, and embed several adversarial networks to realize various category alignments between domains.

### 5.4. Results Analysis

(1) Performance comparison with existing methods.

Table 3 provides a comparison between existing models and our method. Note that the models marked * are reimplemented version because their precision and recall values have not been reported. The model marked + denotes that it contains multiple models, and we only report the best results among them. The results of our models are obtained under the parameter settings that achieve the best results for the development dataset (GCN layers = 3, α = 0.4). From the table, we can see that a model removing adversarial training and only using fixed weights (i.e., CNN+GCN) outperforms the CNN by 2% in terms of macro-F1 score and obtains results that are comparable to those of the DANN. This means that local feature adaptation and non-local feature adaptation are equally important for cross-domain relation extraction.

After adding adversarial training, DANN+CNN achieves results that are comparable to those of the state-of-the-art model (CVAN) in terms of macro-F1. $\mathrm{DANN}+{\mathrm{GCN}}^{2}$ indicates the model that uses only dynamic attention weights; this also improves the baseline DANN model but only achieves similar results to those of DANN+GCN. We assume that dynamic weights cannot capture all word co-occurrence information due to a lack of global statistical knowledge. The ensemble model (HVS) performs the best among the methods from previous works, especially in the bc domain. To illustrate the performance of our model more convincingly, we also compare it with the ensemble model. Our combined model (DANN+GCN+DA) outperforms all the existing models, including the ensemble model (HVS), in terms of macro-F1, and achieves almost all of the best precision and recall performance in the three domains.

(2) Data distribution of source and target domains.

**Figure 7:**
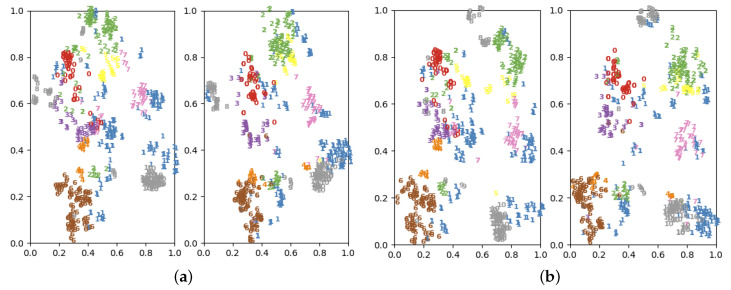
Data distribution under different relation types. (**a**) Original data distribution of source domain (left) and target domain (right). (**b**) Data distribution of source domain (left) and target domain (right) under our method.

In this paper, the source domain dataset bn+nw and the target domain dataset cts are taken as examples. To see the change in data distribution more intuitively, we used the high-dimensional data dimensionality reduction algorithm t-distributed stochastic neighbor embedding (t-SNE) to map the data distribution in two-dimensional space. The data distribution before and after applying our method is shown in Figure 7. As can be seen from Figure 7a,b, after using graph adaptation network, the cts overlapping data is effectively reduced and data distribution is better than the previous data distribution. The t-SNE visualization also indirectly proves the effectiveness of our model.

(3) Performances with different numbers of GCN layers.

**Figure 8:**
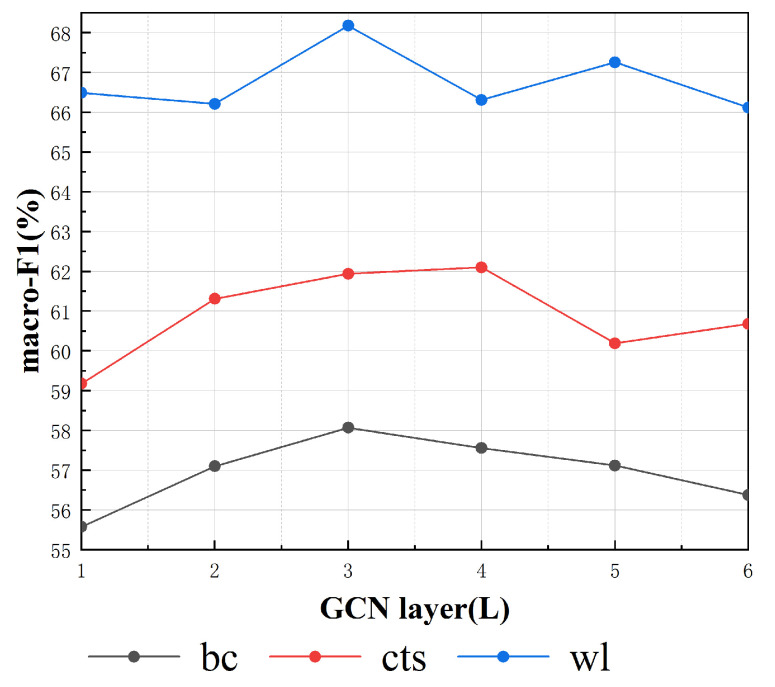
Macro-F1 values with different numbers of GCN layer.

From Equation (14), we know that the number of GCN layers plays an important role in the process of information propagation. In a 1-layer GCN, information only flows between neighbors, so for our tripartite graph, the number of GCN layers is at least 2 so that the information can flow from the source to the target domain. To better verify this intuition and further illustrate the interpretability of our model, we fix the threshold (α) described in Equation (3) as 0.4 and draw Figure 8 to display the relation between the number of GCN layers and the performance (macro-F1) of our model. We report the macro-F1 values for three domains under different numbers of GCN layers L within [1, 2, 3, 4, 5, 6]. It is worth noting that we only draw the line when *L* ≤ 6 because there is no improvement obtained when *L* > 6.

From Figure 8, we can see that the 1-layer GCN has a relatively poor performance on all three domains, but when the number of layers is 3 or 4, the best performance is achieved for all three domains; this illustrates that the 1-layer GCN cannot transfer information from the source domain to the target domain and that a GCN with at least 2 layers can capture the word co-occurrences between the source and target domains. When *L* > 4, the macro-F1 declines to different degrees for all three domains, and we assume that the word representations are distorted due to too many instances of information propagation.

(4) Performances with different thresholds (α).

**Figure 9:**
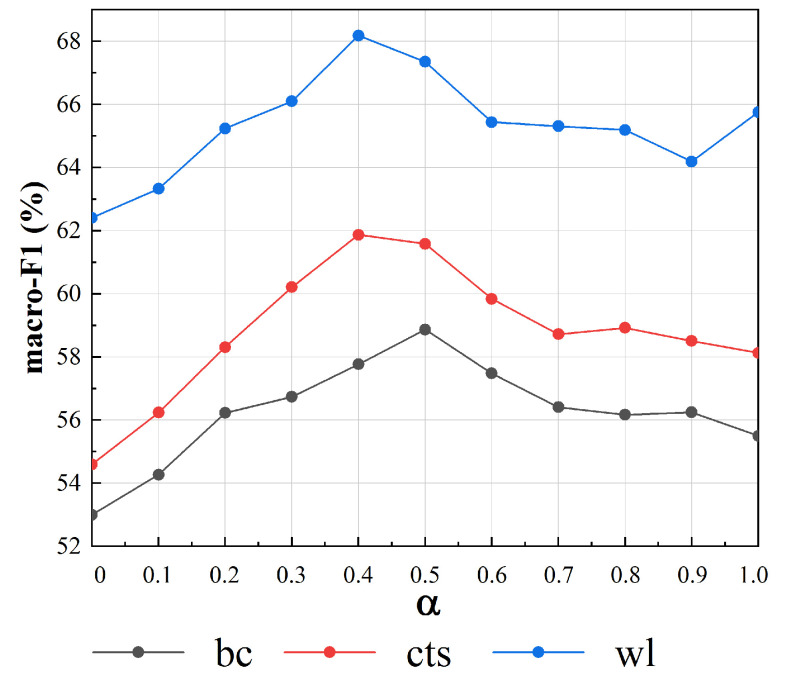
Macro-F1 values with different values of α.

The threshold (α) controls the number of edges whose fixed weights >0. Because the fixed weights of edges are normalized by Equation (6), α varies within the interval [0,1]. From Table 4, we can see that when α is smaller, there are more edges with fixed weights >0 in the graph and vice versa. In particular, when α = 1, there only exist self-loop edges whose fixed weights >0. Figure 9 shows the macro-F1 values with different values of α under the setting “GCN layers = 3”. We can clearly see that when α decreases, macro-F1 suffers from a significant drop. The reason for this phenomenon is that the number of edges with fixed weights >0 increases sharply and therefore inevitably includes more irrelevant information.

The model obtains the best results when α = 0.4 or 0.5, for which the corresponding edge numbers are optimal. When α > 0.5, the performance on all three domains suffers from dramatic decreases. We argue that this is because almost all edges’ fixed weights = 0, and dynamic weights cannot capture complete word co-occurrence information due to a lack of global statistical knowledge. When α is in the interval [0.6, 1], the macro-F1 value remains stable because the graph has a few edges with fixed weights >0. The model performance under different values of α illustrates that simply increasing the number of edges indefinitely is not ideal. The use of a proper number of edges can not only yield better performance but also speed up calculations.

(5) Effects of the GCN and dynamic weights.

**Figure 10:**
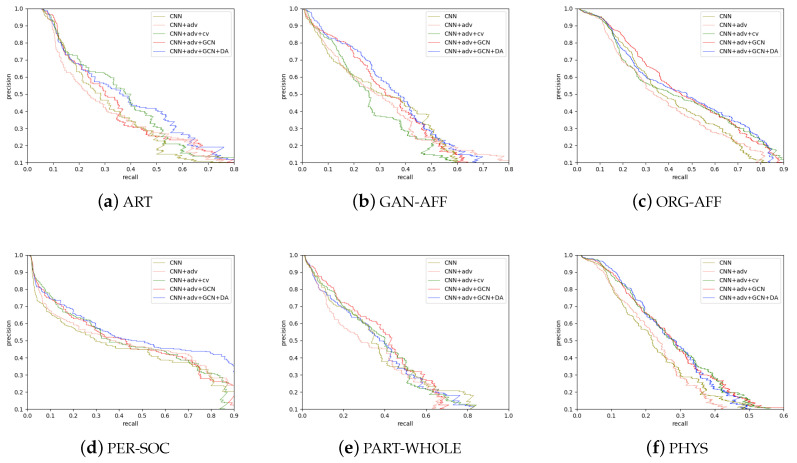
Precision/recall curves under different relation types.

To illustrate the effectiveness of the GCN and the use of dynamic attention weights, we draw the precision-recall curves for every relation type in Figure 10. The values of precision and recall are averaged across all domains. CNN+adv is the reimplemented version of the DANN [11], +cv refers to adding cross-view training, and DA refers to the dynamic attention weights we proposed. From Figure 10, we can see the following: (1) Only using the CNN to perform cross-domain relation extraction is far from enough, and it almost achieves the worst performance for all 6 relations, indicating that it is worth trying domain adaptation on this dataset. (2) The performance of CNN+adv and CNN+adv+cv are better than that obtained when only using the CNN, but CNN+adv+cv is significantly worse than the CNN under the relation GAN-AFF, and CNN+adv is also worse than the CNN under the relation ORG-AFF. We find that the GAN-AFF and ORG-AFF relations are similar and have the most subtypes, so these two relations provide a strong challenge for our model. (3) CNN+adv+GCN yields large performance increases for GAN-AFF and ORG-AFF, and the curves are almost all above the curve of CNN+adv.

This illustrates the effects of the GCN layer. When adding DA to the GCN layer, the improvement is highly obvious, especially under the PER-SOC relation; specifically, there is a 10% improvement in precision when recall >0.7 in Figure 10e. This verifies that DA can compensate for the weakness of fixed weights and consider many words’ connections. For a quantitative analysis of the effects of each model, we average the AUCs under different relations for every method, and these are shown in Table 5. It can be clearly seen that GCN+DA improves the baseline by a large margin (by 4% compared with CNN+adv, by 7% compared with the CNN).

### 5.5. Case Study

**Figure 11:**
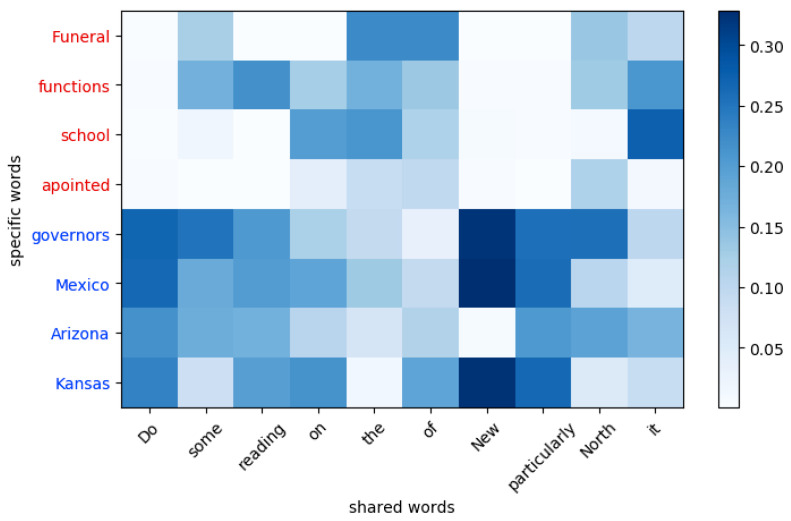
Graph edge weights visualization.

An example of graph weight visualization is shown in Figure 11. The *x*-axis denotes the shared words of different domain sentences, the red part of the *y*-axis denotes source-specific words, and the blue part denotes target-specific words. Each pixel corresponds to the weight $W_{ij}$ of the *i*-th shared word and the *j*-th specific word described in Equation (11), and the deeper the colour is, the greater the $W_{ij}$. All the weights corresponding to the *i*-th shared word are normalized. The colors between the word *new* and some target-specific words such as *Mexico* and *Kansas* are deepest because their fixed weights are large according to Equation (7). In addition, some specific words with strong domain relevance, such as *Funeral* and *School*, also have deep colors, while the colors of words with weak domain relevance, such as *functions* and *apointed*, are relatively shallow. These facts illustrate that the dynamic weight mechanism pays more attention to aligning specific words with stronger domain relevance; the sole use of fixed weights cannot be achieved.

We also present some typical examples in Table 6 that demonstrate the effectiveness of our model. In some cases, such as the first two samples, traditional models and our model can all correctly predict the labels. However, when the domain-specific words have strong domain relevance or account for a high proportion of the sentence, traditional models mistake all the labels for “None” (as in the last three examples) because negative samples account for a large proportion (Table 1). Our model correctly distinguishes between negative and positive samples and predicts true labels in all the above cases. We argue that traditional models only capture shared features based on local information, i.e., words in sequential order; therefore, when the proportion of domain-specific words increases, the number of shared features decreases. In addition, the shared feature extractor may be unable to capture shared features because these specific words have strong domain relevance; in other words, the domain discriminator still has the ability to determine which domain a given sample comes from. Through the GCN layer we proposed, these target-specific words are aligned with source-specific words, so the interference of domain-specific features is reduced.

## 6. Conclusions

In this article, a novel graph adaptation network for cross-domain relation extraction is proposed. First, a novel graph adaptation network is constructed to align domain-specific features. The model aligns domain-specific features through applying graph convolutions on a source-shared-target words tripartite graph. Secondly, unlike the traditional methods only adapting local or sequential features, our model adapts local and non-local features jointly to improve the performance of cross-domain relation extraction. Finally, a cross-domain relation extraction model is constructed by inputting the fused all features into softmax.

In addition, unlike the traditional graph convolutional network, in order to compensate for the limitation of fixed weight, a dynamic attention weight is combined together with fixed weight. To transmit useful information more effectively, we retain valuable edges based on their edge weights. Experiments on the three domains of ACE2005 datasets verified the effectiveness of GCN layer and dynamic attention weight, which achieve state-of-the-art on all three domains.

We will further explore the cross-domain relation extraction when a few labeled examples can be explored or new relations appearing in the target domain. In addition, we hope our work can be applied to other domain adaptation tasks in the future.

## Figures and Tables

**Table 1:** ACE2005 dataset statistics.

Domain	Total	#Entity Type	Negative Relation Rate
bn+nw	43,497	43	0.916
bc_dev	7004	33	0.912
bc_test	8083	33	0.911
cts	15,903	40	0.961
wl	13,882	30	0.949

**Table 2:** Parameters settings of our model. *dd* and *rc* refer to the domain discriminator and relation classifier, respectively.

Parameters	Settings
Word embedding dimensions	300
Dt/ch/et/ep embedding dimensions	50
kernel size	[2, 3, 4, 5]
Number of kernels	150
Hidden dimension of dd, rc	300
Hidden dimension of GCN layer	100
Learning rate	0.001
Dropout rate	0.5
Batch size	128
Interval of gradient clipping	[−1, 1]
Windows size of words co-occrrences	[20]

**Table 3:** Precision (P), recall (R) and macro-F1 (%) comparison with existing methods on three domains.

Models	bc			cts			wl		
	P	R	F1	P	R	F1	P	R	F1
BRNN	65.23	61.06	63.07	66.15	49.26	56.47	55.91	51.56	53.65
CNN	65.62	61.06	63.26	65.92	48.12	55.63	54.14	53.68	53.91
Log-linear	68.44	50.07	57.83	73.62	41.57	53.14	54.14	47.31	53.06
HVS ${}^{+}$	70.40	63.84	66.96	65.91	52.21	58.26	58.81	55.81	57.27
FCM	66.56	57.86	61.9	65.62	44.35	52.93	57.80	44.62	50.36
HFCM	74.39	55.35	63.48	74.53	45.01	56.12	65.63	47.59	55.17
LRFCM	65.1	54.7	59.4	-	-	-	-	-	-
GSN ${}^{*}$	66.78	64.12	65.42	68.89	48.71	57.07	59.32	52.91	55.93
CVAN	73.95	60.92	66.81	70.43	53.61	60.18	59.2	56.13	57.62
DANN ${}^{*}$	70.21	59.36	64.33	71.43	47.72	57.2	58.37	54.26	56.24
AGGCN	−	−	63.47	−	−	59.70	−	−	56.50
MADA-weight	−	−	65.86	−	−	56.33	−	−	56.10
CNN+GCN	69.82	61.46	65.37	64.57	52.63	57.99	62.59	50.67	56
DANN+GCN	75.12	60.46	67	64.67	57.35	60.79	60.52	54.8	57.52
DANN+GCN${}^{2}$	70.56	63.28	66.72	60.08	58.57	59.32	63.12	53.08	57.67
DANN+GCN+DA	72.55	64.31	68.18	65.4	59.1	62.1	65.93	53.17	58.87

Model marked with ${}^{+}$ contains multiple models, and only the best results among them are reported. Models marked with ${}^{*}$ present reimplemented versions, as their precision and recall values have not been reported.

**Table 4:** The total and average number of edges whose fixed weights >0 under different values of α. The total number of edges is calculated based on the combination of all source and target domain data. The average edge number indicates the average number of edges in one sentence. None of the statistics include self-loop edges.

α	#Total Edge Number (Average Edge Number)		
	bc	cts	wl
0	392,552 (348)	401,541 (360)	454,692 (321)
0.1	288,202 (172)	290,678 (241)	344,694 (221)
0.2	191,256 (129)	191,858 (154)	242,238 (144)
0.3	110,734 (78)	106,068 (85)	150,442 (80)
0.4	48,838 (30)	45,870 (32)	73,212 (51)
0.5	15,352 (10)	14,342 (9)	25,184 (19)
0.6	4220 (4)	3952 (3)	6632 (4)
0.7	1012 (2)	954 (2)	1602 (2)
0.8	262 (1)	272 (2)	344 (1)
0.9	6 (0.05)	44 (0.3)	18 (0.2)
1.0	0 (0)	0 (0)	0 (0)

**Table 5:** AUCs of different methods.

Models	CNN	CNN + adv	CNN + adv + cv	CNN + adv + GCN	CNN + adv + GCN + DA
AUC	0.345	0.378	0.385	0.397	0.41

**Table 6:** Some predictions of typical samples comparison. We use DANN and CVAN as traditional models. The words with bold are domain-specific.

Predict Label		Examples
Traditional	Ours	
ART *√*	ART *√*	In which, the state of Israel **buys** many **expensive** military weapons, which is used to **oppress** and to kill a lot of Palestinians.
ORG-AFF *√*	ORG-AFF*√*	It could be done so **easily** because most small **towns** are protected by a small town police force.
None ×	PHYS *√*	well, my sister usually comes in from **Ohio** because it’s not that far like–two hundred **fifty** miles or something, and she’ll come in for **Thanksgiving**.
None ×	GAN-AFF *√*	That’s in **Fairmont**, West **Virginia**, it’s like—oh, between **Charleston** and **Pittsburgh**.
None ×	PER-SOC *√*	My **brother-in-law** still lives in the city but we were **Long Island** people.
